# The impact of injury definitions on measures of injury occurrence in classical music students: a prospective cohort study

**DOI:** 10.1186/s12891-020-03490-0

**Published:** 2020-08-11

**Authors:** Suze Steemers, Marienke van Middelkoop, Gideon G. de Boks, Rogier M. van Rijn, Sita M. A. Bierma-Zeinstra, Janine H. Stubbe

**Affiliations:** 1 Codarts Rotterdam, University of the Arts, Kruisplein 26, 3012 CC Rotterdam, The Netherlands; 2 Department of General Practice, Erasmus MC Medical University Center, Wytemaweg 80, 3015 CN Rotterdam, The Netherlands; 3 Performing artist and Athlete Research Lab (PEARL), Kruisplein 26, 3012 CC Rotterdam, The Netherlands; 4 Rotterdam Arts and Sciences Lab (RASL), Nieuwemarkt 1A, 3011 HP Rotterdam, The Netherlands

**Keywords:** Music students, Injury, Musculoskeletal complaints, Time-loss, Medical attention

## Abstract

**Background:**

Multiple definitions are used to investigate injuries in musicians, resulting in a wide range of prevalence rates. The aim of this study is to establish the impact of different injury definitions on the prevalence of injuries and disability in classical music students. Moreover, the practical implications of using different injury definitions are considered.

**Methods:**

A prospective cohort study among first-year classical music students was performed using bi-monthly questionnaires focusing on injuries. Three injury definitions were used: 1) all MSK complaints injury (any MSK complaint resulting in a VAS pain score > 0 in the past eight weeks), 2) medical attention injury (any MSK complaint that resulted in a student consulting a health provider in the past eight weeks), 3) time-loss injury (any MSK complaint that resulted in partly/completely missing music classes or activities in the past eight weeks). For all injury definitions prevalences were calculated and compared. Furthermore, the Disabilities of the Arm, Shoulder and Hand performing arts module (DASH-pa) was used to calculate disability scores for all three injury definitions.

**Results:**

Twenty-nine classical music students participated in the current study. The total response rate over one academic year was 85.3%. One year prevalences of all MSK complaints, medical attention and time-loss were 96.6, 17.2 and 13.8% respectively. The bi-monthly prevalences ranged from 74.1 to 96.0% for all MSK complaints, from 5.6 to 11.5% for medical attention injuries and from 0 to 11.5% for time loss injuries. Scores on the DASH-pa ranged from 15.6 to 26.9 for MSK complaints, 33.3 to 50 for medical attention and 47.9 to 62.5 for time-loss injuries.

**Conclusion:**

The choice of injury definition is a critical factor affecting the outcome of music injury surveillance studies. To reach a consensus, it is therefore important to consider the different injury definitions depending on the goal of the injury surveillance and the practical implications.

## Background

Musicians are at risk for injuries during their career. They usually experience high musculoskeletal (MSK) and psychosocial demands within their profession [1–3]. Professional musicians practice daily for many hours in which they constantly repeat physical movements in ergonomically unfavorable postures to reach an elite level [1]. Beginning in their music education, music students experience more MSK complaints compared to age-matched medical students [4]. Pain in the shoulders, back and neck are the most frequently mentioned in this population [1, 4, 5]. To develop effective preventive measurements, it is essential to establish the extent of the injury [6].

In studies that have focused on injuries in musicians, prevalence and incidence rates vary considerably. A recent systematic review found varying percentages between 9 to 68% for MSK complaints in professional musicians [7]. This large range in the prevalence of injuries may be explained by the use of different definitions in the included studies. Among others, Playing-Related Musculoskeletal Disorders (PRMD), ‘pain’ and questions from the Nordic MSK Questionnaire were used to define MSK complaints [8–10]. The need for an operational definition of PRMD was already addressed by Zaza and colleagues [11]. However, even after establishing the definition for PRMD, which is to date one of the most commonly used terms in musicians health research, it was frequently used as an umbrella term for different musculoskeletal disorders in various studies [7, 12]. Furthermore, definitions with both playing related and non-playing related MSK complaints were included in these reviews. Yet, the impact of different measures and definitions on the outcomes in classical music students has not been studied.

The influence of different injury definitions on their outcome estimates has been studied across different kinds of sports [13, 14]. Consensus statements were published to establish definitions and reporting standards for injury surveillance studies [13, 15]. Definitions were proposed to establish when an injury was a time-loss or a medical attention injury; injuries defined as time-loss describe injuries that result in ‘a player being unable to take a full part in future sports training or match play’. Whereas, injuries defined as medical attention are the result of ‘athletes receiving medical attention’. Medical attention refers to ‘the assessment of an athlete’s medical condition by a qualified medical/healthcare practitioner’ [13, 14]. A study by Clarsen and Bahr (2014) reviewed consensus-based sports injury definitions and concluded that there is no, single ‘one-size-fits-all’ injury definition. The authors proposed three widely-used injury definitions instead: [1] all complaints, [2] medical attention and [3] time-loss, in which the all complaints definition focuses on ‘registration of all medical problems, including those that do not lead to medical attention’ [16]. Therefore, in sports research, it is recommended to choose one of these definitions, taking into account the context, aim and practical implications of the injury surveillance e.g. duration, setting, type of surveillance and the goal of data collection [16].

In contrast to sports, there are hardly any studies investigating the influence of injury definitions among performing artists. To our knowledge only one study was conducted within dance [17]. Similar to musicians, dancers usually have a high prevalence of overuse injuries and are inclined to continue performing despite pain complaints [17, 18]. Kenny et al. concluded that the definitions of medical attention and time-loss may underestimate the prevalence and incidence rates of overuse injuries within a population of dancers compared to an all complaints definition [17]. Therefore, other injury definitions are also needed to facilitate the prevention of injuries and may result in the earlier detection of injuries within performing artists [6].

A number of studies have already shown that the choice of injury definition within sports and dance could influence the outcome estimates [13, 16, 17], but no study so far has focused on this topic in a population of classical music students. Therefore, the aim of the current study is to determine the impact of three different injury definitions on the prevalence of injury and disability within classical music students. In addition, the practical implications of using these three definitions will be discussed.

## Methods

### Design and participants

This prospective cohort study was performed among first-year classical music students at Codarts University of the Arts, Rotterdam, The Netherlands. All first year students following the Bachelor of Classical Music were invited to participate and the participation was offered as part of an educational program focusing on health-related subjects in classical music. All participants were informed about the procedure and provided informed consent. The study was approved by the Medical Ethics Committee (MEC-2019-0163) of the Erasmus MC University Medical Center Rotterdam, The Netherlands.

### Procedure

At the start of the academic year, all participating students were asked to complete an online intake questionnaire using the Performing artist and Athlete Health Monitor (PAHM) [19]. PAHM is a web-based system, developed to monitor physical and mental health in athletes, performing arts students and performing artists [19, 20]. The intake questionnaire included items on age, sex, body weight, height and previous injuries (injuries lasting at least one week in the previous year). In addition, all students were asked about their main subject (instrument), playing history (years of playing), their break behavior (minutes), warm-up (yes/no, with or without instrument) and estimated playing hours per week. During the academic year, bi-monthly questionnaires were sent to all participating students. These follow-up questionnaires included questions on the occurrence of any MSK complaints in the past eight weeks on a Visual Analogue Scale (VAS) (0–100; 0 – no pain; 100 – worst pain you can imagine) [9], time-loss (number of days partly/completely missed music activities due to a musculoskeletal problem), and contact with a health provider (yes/no). To establish the extent of the disability when playing a musical instrument, the Disabilities of the Arm, Shoulder and Hand performing arts module (DASH-pa) was used [21]. The DASH-pa is an optional module of the DASH that has shown good internal consistency and discriminative validity between music students with and without PRMD [22]. The DASH-pa consists of four items (DASH1, DASH2, DASH3, DASH4), with scores from 0 (not disabled) to 100 (most severe disability), which together form a DASH-pa sum score using a specific calculation. Non-responders received two reminder e-mails within a week and were also reminded about the questionnaire in person during classes.

### Injury registration

In agreement with the literature by Clarsen and Bahr on sports injury definitions [16], we used the three sports injury definitions and slightly modified these to better fit our specific target population (i.e. classical music students):

- all MSK complaints injury: any MSK complaint resulting in a VAS pain score > 0 in the past eight weeks.

- medical attention injury: any MSK complaint that resulted in a student consulting a health provider in the past eight weeks.

-time-loss injury: any MSK complaint that resulted in partly/completely missing music classes or activities in the past eight weeks.

### Statistical methods

Statistical analyses were conducted using SPSS version 24. Baseline characteristics were calculated with descriptive statistics using means and standard deviations (SD) or numbers and percentages (%). Prevalences for one academic year were calculated by dividing the number of students that reported at least one injury (according to the three injury definitions) during the academic year by the number of respondents in that same period [23]. Confidence intervals (CI) for all three definitions were compared.

Prevalences for a two-month time period were calculated by dividing the number of students that reported at least one injury (according to the three injury definitions) during those two months by the number of respondents in that same period [23].

For the Visual Analogue Scale, the bi-monthly mean scores and ranges were included. Categories were used to establish a mild (≤ 30), moderate (31–69) or severe (≥ 70) level of MSK complaints [24].

Following the scoring instructions by the Institute for Work & Health [25], the DASH-pa sum scores were established by using the following calculation: ((((DASH1 + DASH2 + DASH3 + DASH4) / 4) - 1) * 25). The scores were calculated for the three injury definitions separately by using the sum scores of all students who indicated one of the three injury definitions. So for example: the average of all DASH-pa sum scores of all students seeking medical treatment formed the DASH-pa score for medical attention.

## Results

Baseline characteristics of the participating students are shown in Table 1. A total of 31 students were enrolled in the first year Bachelor of Classical Music program at Codarts. All students gave consent to participate and 29 students were included. Two students only filled in the intake questionnaire and were therefore excluded from the analyses. In total, 116 questionnaires were sent to the students and 99 were completed, resulting in a total response rate of 85.3%.

**Table 1:** Baseline characteristics shown as mean (±SD) or number (percentage)

	Mean or number
N	29
gender (female)	16 (55.2%)
age (years)	20.85 (±2.6)
instrument (main subject)	
violin	6 (20.7%)
double bass	4 (13.8%)
voice	3 (10.3%)
cello	2 (6.9%)
percussion	2 (6.9%)
trumpet	2 (6.9%)
bass trombone	2 (6.9%)
trombone	2 (6.9%)
other	6 (20.7%)

### All MSK complaints injury

**Fig. 1:**
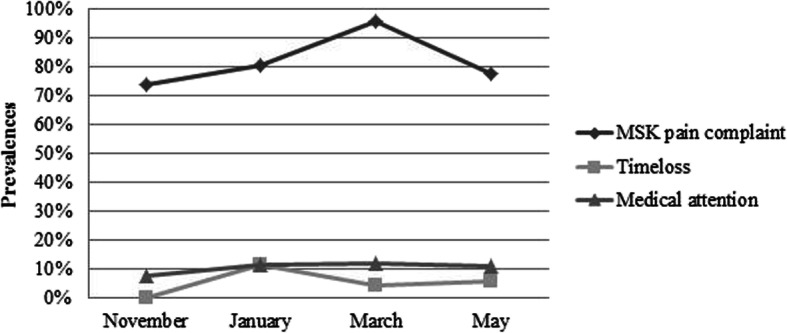
Bi-monthly prevalences of MSK complaints, time-loss and medical attention definitions

**Fig. 2:**
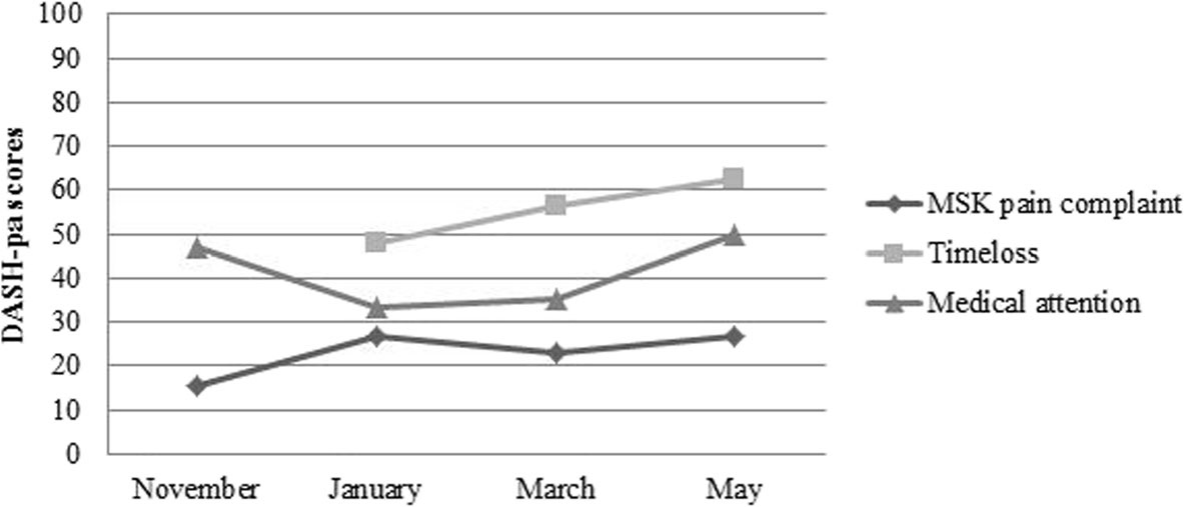
Bi-monthly DASH-pa scores for MSK complaints, time-loss and medical attention injuries

In total, 28 out of 29 students reported at least one MSK complaint (VAS > 0) during the academic year, resulting in a one year prevalence of 96.6% (95% CI: 0.899, 1.032). The bi-monthly prevalences ranged from 74.1 to 96.0% (Fig. 1). The average scores on the VAS ranged from 15.63 to 29.83 (Table 2). In all periods (November until May) most students reported mild MSK complaints, ranging from 50 to 85.2%. Bi-monthly DASH-pa scores for all students reporting a MSK complaint ranged from 15.6 to 26.9 (Fig. 2).

**Table 2:** Bi-monthly scores on the VAS

Period	Mean (range)	Severity of complaints		
		mild	moderate	severe
November	15.63 (0–58)	85.2%	14.8%	0%
January	29.83 (0–80)	50%	46.2%	3.8%
March	24.80 (0–79)	72.0%	24.0%	4.0%
May	24.78 (0–66)	66.7%	33.3%	0%

### Medical attention injury

In total, five out of 29 students visited a health provider throughout the academic year, resulting in a one-year prevalence of medical attention injuries of 17.2% (95% CI: 0.035, 0.31). The students’ main subjects were double bass (*n* = 2), violin, trombone and vocals (Table 3). The bi-monthly prevalences for medical attention ranged from 7.4 to 12.0%. (Fig. 1). The five students indicated that they had visited a physiotherapist or speech therapist (six physio treatments, three speech therapist treatments and one treatment by both physio and speech therapist) during one year. DASH-pa scores for all students reporting a medical attention injury ranged from 33.3–50 (Fig. 2).

**Table 3:** Main subjects of students with medical attention and students with time-loss injuries

	Medical attention (N)	Time-loss injury (N)
Double bass	2	3
Violin	1	
Vocals	1	
Trombone	1	
Bass trombone		1

### Time-loss injuries

In total, four out of 29 students reported to have partly/completely missed music activities for at least a day due to a MSK complaint during one academic year, resulting in a prevalence of 13.8% (95% CI: 0.012, 0.263). The students’ main subjects were double bass (*n* = 3) and bass trombone (Table 3). The bi-monthly prevalences of time-loss injuries ranged from 0 to 11.5% (Fig. 1). The average number of days missed because of a MSK complaint was 2.6 (SD 1.8). Two students who reported a time-loss injury received medical care at the same time. Both those students played double bass. DASH-pa scores for all students reporting a time-loss injury ranged from 47.9 to 62.5 (Fig. 2).

**Fig. 3:**
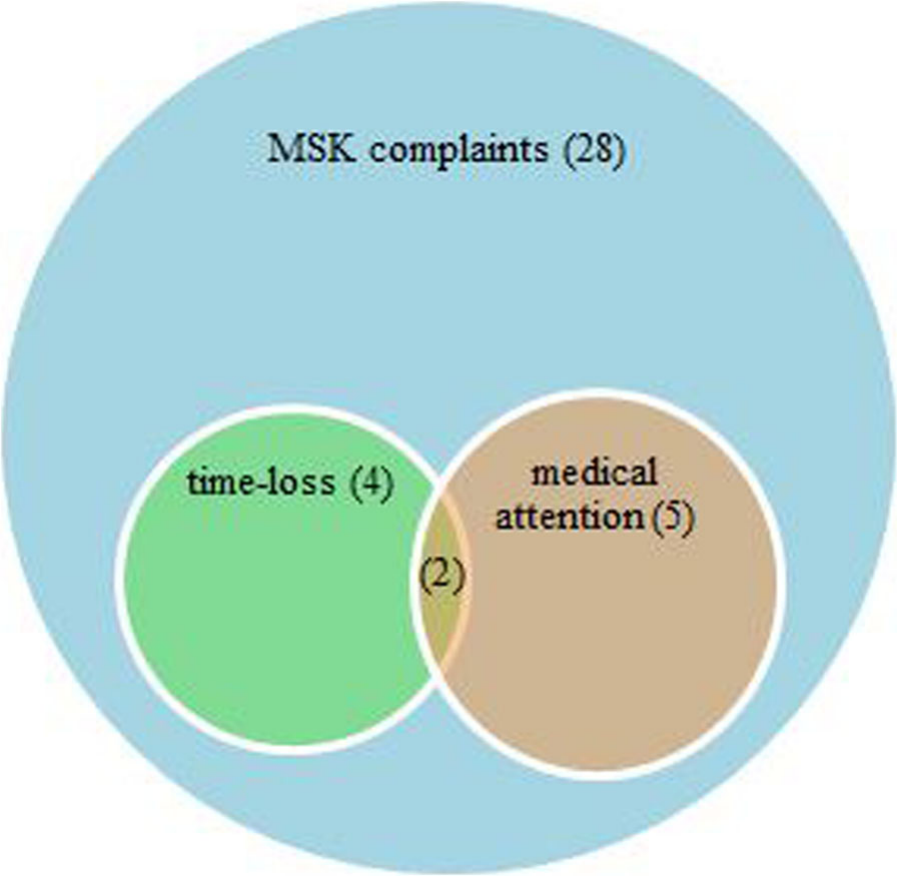
Distribution and overlap of different injury definitions in classical music students

Figure 3 shows the distribution and overlap of the three different injury definitions over one academic year. The figure represents all students with a MSK complaint, *n* = 28 (100%), in the previous year and shows which of those complaints resulted in a medical attention injury, *n* = 3 (10.7%), time-loss injury, *n* = 2 (7.1%) or both a medical attention and time-loss injury, *n* = 2 (7.1%).

## Discussion

The aim of this study was to determine the impact of different injury definitions on injury prevalences and disability within classical music students. Results from this study show that injury definition affects outcomes of music injury surveillance studies. The one year prevalences for MSK complaints, medical attention and time loss injuries were 96.6, 17.2 and 13.8%, respectively. Thus, there were significant differences between the one-year prevalences across the definitions. The 95% Confidence Intervals for MSK complaints, medical attention and time-loss were 0.899,1.032; 0.035, 0.31 and 0.012, 0.263, respectively.

Except for one, all students experienced a MSK complaint throughout the academic year. In comparison, the one year prevalences for medical attention and time-loss injuries were much lower, i.e. 17.2 and 13.8% respectively. These findings are in line with studies conducted within sports and dance [15, 17, 26] in which the ‘all complaints’ definition resulted in higher injury prevalences compared to the use of medical attention or time-loss definitions. This implies that injury definitions do significantly affect the outcome of injury surveillance in classical music students. Therefore, it seems essential to reach a consensus on injury definitions in this population.

The definition of MSK complaints captures more overuse injuries than acute injuries and does not exclude minor complaints [26]. Within the sports literature, the use of different definitions has already been studied by Bahr [15]. In this study, two different recording systems for measuring volleyball injuries were compared resulting in much lower prevalence rates for time-loss injuries than all complaint injuries. The results in the current study with a much higher prevalence for MSK complaints compared to time loss, suggest the same. Most of the indicated MSK complaints were mild, however could become overuse injuries if not taken seriously or treated. In order to get insight into these minor complaints as well, early detection is needed to facilitate prevention and treatment, if necessary. The all MSK complaints definition seems the most appropriate to reflect the total impact of injury burden in musicians.

In contrast, the medical attention definition seems to focus more on acute injuries than on overuse injuries [26]. Within Codarts, a wide variety of health specialists are available in the extensive student support program, Student Life, e.g. physiotherapists, a dietician, a mental coach, a psychologist and a speech therapist. These professionals all have much experience in supporting and treating performing artists and most of them have a background in performing arts. Despite this support system, the percentage of medical attention injuries is relatively low, which is in agreement with earlier studies that showed that musicians may not always seek medical treatment because they fear the advice to reduce playing the full musical program, in concerts or at rehearsals [18]. This is supported by Guptill et al. (2000) who found that 96% of college music students had experienced pain symptoms as a result of playing their instruments, before seeking treatment [27]. Moreover, Ioannou and colleagues. (2015) found that asking for medical care is considered to be a taboo among music students [28]. Whenever MSK complaints arise, students seem to primarily go to their teacher for help, at least when these complaints are playing related. Therefore the medical attention definition may underestimate the actual injury burden in this population of classical music students.

For the management staff of orchestras and conservatoires the time-loss definition may be most suitable in gaining insight into the employability of musicians for a rehearsal or concert. The results of the current study show a large discrepancy between the total amount of MSK complaints and the total amount of time-loss injuries. Within the orchestra culture, injuries are often concealed due to negative perceptions because of job insecurities, injury stigmatization and fear of judgement [2, 18]. Therefore, it can be expected that musicians and music students experience quite a lot of pain before they decide to stop playing due to an injury. This is in agreement with the earlier mentioned study by Ioannou and colleagues (2015) in which 25% of the students who experienced pain indicated that they always continued playing. Half of the affected students mentioned that they continued sometimes [28]. This may lead to a worse prognosis of their complaint. The time-loss injury definition seems very practical to use, however it does not seem suitable for understanding the overall injury burden.

### Interaction between definitions

Bahr and Clarsen proposed a model that represents interactions between various injury definitions for sports [16]. It is assumed that all time-loss injuries are medical attention injuries as well, indicating that athletes visit a health provider when time-loss injuries occur. It is also assumed that all medical attention injuries are part of the total injury burden, measured with the ‘all complaints’ definition. In our study focusing on classical music students, this specific model does not seem fully applicable. Figure 3 shows that not all students reporting a time-loss injury also indicate a medical attention injury. This could be explained by the cultural norm in the performing arts as noted before [17, 18]. More insight into the culture of the performing arts can provide practical implications for prevention. Qualitative studies can provide insight into the underlying causes of the cultural norm. Concerning the changes in the interaction model for a population of musicians, prospective studies with a larger population should be conducted to confirm these.

### Strengths & Limitations

This is the first study investigating the influence of the three injury definitions proposed by Clarsen et al. [16] on prevalences in a group of classical music students. A strength of our prospective cohort study is the response rate of 85%, which is considered high in this population. One of the advantages of the current study is that filling in the bi-monthly questionnaire was part of an educational program within the curriculum. This stimulated the participants to fill in the questionnaire. Also, this is one of the first studies using a prospective cohort design which gives us the possibility to monitor music students’ complaints over a longer period of time. Furthermore, the DASH-pa module used in the current study, was previously examined by Baadjou and colleagues (2017) and showed good discriminative validity, good internal consistency and moderate construct validity in a population of music students [22].

However, there are some limitations. First of all, the sample size in the current study is small which makes it difficult to draw firm conclusions. Future research should include a larger sample size in order to overcome this issue. The few students who suffered from a time-loss injury all played a large instrument. We suggest to elaborate more on this in future research, using a larger sample to see whether students playing larger instruments actually suffer more from time-loss injuries.

Secondly, we did not explicitly ask about the occurrence of Playing-Related Musculoskeletal Disorders (PRMD) and were therefore not able to calculate the prevalence of this type of injury. The original definition developed by Zaza and colleagues (1998) is: ‘pain, weakness, numbness, tingling or other symptoms that interfere with (their) ability to play (their) instrument at the level (they) are accustomed to’ [11]. Using PRMD as an injury definition can be interesting especially for musicians, conservatoires and music healthcare specialists, in order to focus on prevention within the specific environment of musicians. Additionally, information about the location of PRMD might be useful to develop prevention programs focusing on different instrument groups.

Moreover, we used a period of eight weeks to monitor the students. While monitoring on a monthly basis provides more information and decreases recall bias, due to scheduling limitations it was not possible to implement a monthly questionnaire in the curriculum structure.

## Conclusion

In conclusion, the use of three different definitions of injuries in classical music students shows a considerable impact on the prevalence of injury and the disability experienced. The highest prevalences were found for all MSK complaints (96.6%), followed by those students requiring medical attention (17.2%) and time-loss injuries (13.8%). The three injury definitions also resulted in differences in the ranges of disability scores, with the highest scores for time-loss injuries. These results suggest that the choice of injury definition is a critical factor affecting the outcome of music injury surveillance studies. To allow consistent measurements of MSK complaints in inevitably small populations of musicians that need to be pooled, we would like to emphasize that consensus needs to be reached regarding injury definition in this specific target group.

## Data Availability

The datasets used and/or analyzed during the current study are not publicly available due to the fact that the data are not anonymous but pseudo-anonymous and contain medical data. The datasets are available from the corresponding author on reasonable request.

## Abbreviations

MSK: Musculoskeletal
DASH-pa: Disability of the Arm, Shoulder and Hand performing arts
PRMD: Playing-Related Musculoskeletal Disorders
Nordic MSK Questionnaire: Nordic Musculoskeletal Questionnaire
MEC: Medical Ethics Committee
PAHM: Performing artist and Athlete Health Monitor
VAS: Visual Analogue Scale
SD: Standard Deviations
CI: Confidence Intervals
